# Advancements in Cellulose-Based Materials for CO_2_ Capture and Conversion

**DOI:** 10.3390/polym17070848

**Published:** 2025-03-22

**Authors:** Niranjan Patra, Prathipati Ramesh, Ștefan Țălu

**Affiliations:** 1 Department of Chemistry, Koneru Lakshmaiah Education Foundation, Greenfield, Vaddeswaram 522502, Andhra Pradesh, India; 2 The Directorate of Research, Development and Innovation Management (DMCDI), The Technical University of Cluj-Napoca, Constantin Daicoviciu Street, no. 15, 400020 Cluj-Napoca, Cluj County, Romania; stefan.talu@auto.utcluj.ro

**Keywords:** sustainable materials, cellulose, CO_2_ adsorption, CO_2_ conversion

## Abstract

This study explores the recent advances of cellulose-based materials in the context of carbon capture and conversion amid the global imperative to reduce CO_2_emissions. The review emphasizes the critical importance of selecting suitable materials for establishing a robust and secure carbon capture technology. From elucidating celluloses’ molecular structure and unique properties to detailing the advancements in CO_2_ capture technologies, the narrative provides a comprehensive understanding of the intricate interplay between cellulose and sustainable CO_2_ management. The exploration extends to the design and synthesis of cellulose-based adsorbents, challenges in implementation, showcasing emerging trends and potential breakthroughs. Emphasizing the significance of cellulose in the circular carbon economy, this review serves as a beacon for interdisciplinary collaboration, urging further research and implementation for a greener and more sustainable future. A comprehensive overview of recent developments on cellulose-based aerogels, films, composites, and solid adsorbents in the field of carbon capture. It further elucidates the research mechanisms involved in utilizing cellulose-based materials to convert CO_2_ into formic acid, methanol, carbonate, and CO, offering detailed insights. The review concludes by addressing the challenges and key issues associated with cellulose-based materials in the context of carbon capture and utilization.

## 1. Introduction

The specter of climate change, propelled by escalating CO_2_ emissions, resonates with a gravity that transcends geographical and disciplinary boundaries. The scientific consensus, embodied in reports such as the Intergovernmental Panel on Climate Change (IPCC) assessments, underscores the imperative to curtail CO_2_ emissions to mitigate the severe consequences of global warming [1]. The atmospheric concentration of CO_2_, having surpassed 400 parts per million, has become emblematic of an era characterized by unprecedented challenges to the Earth’s climate stability. In the pursuit of mitigating CO_2_ emissions, the clarion call for sustainable solutions has become a driving force. The coupling of economic growth with environmental stewardship has emerged as a non-negotiable paradigm shift, resonating in policy initiatives and corporate strategies alike. The need to align technological advancements with ecological resilience demands an intricate dance of innovation and responsibility, emphasizing the urgency of transitioning towards sustainable practices [2].

Solid, liquid, and gel-like substances are among the many materials recognized as efficient CO_2_ adsorbents and catalysts. Improvements in selectivity, impurity tolerance, regeneration capacity, and overall capacity for CO_2_ adsorption and conversion have been the primary goals of carbon capture material research [2,3,4,5,6,7,8]. For CO_2_ capture, traditional adsorbents like carbon, silica, and zeolite have been studied extensively [7]. New adsorbents like metal-organic frameworks, hydrotalcites, amine-based adsorbents, polymers, and metal oxides are also under investigation [8,9,10,11,12,13].

Cellulose is an excellent biopolymer abundantly woven into the fabric of terrestrial plant cell walls. Its molecular architecture, comprised of linear chains of glucose linked by β-1,4-glycosidic bonds, imparts cellulose with unparalleled structural integrity and renewability [14]. The abundance of cellulose in nature, derived from diverse sources such as wood, cotton, and agricultural residues, positions it as an ideal candidate for sustainable material synthesis [15]. Within the crucible of materials innovation, cellulose-based materials emerge as protagonists in the narrative of CO_2_ capture and utilization. The porous structure of cellulose, coupled with its chemical functionality, providesit with an innate capacity to sequester CO_2_ from diverse sources, ranging from industrial flue gases to ambient air [16]. Moreover, the catalytic potential of cellulose derivatives opens avenues for transforming captured CO_2_ into value-added products, thereby forging a circular economy that transcends the traditional boundaries of waste and resources [17].

Providing a thorough evaluation of cellulose-based materials for CO_2_ capture and conversion, this review highlights their potential as substitutes for traditional adsorbents and catalytic systems. The distinct benefits of cellulose, such as its renewable nature, adjustable surface chemistry, and ecological sustainability, have been largely unexplored in carbon capture research, in contrast to metal-organic frameworks (MOFs), zeolites, and amine-based sorbents. This study provides a comprehensive analysis of the latest developments in cellulose-based adsorbents, focusing on the structural changes, functionalization methods, and performance improvements made to these materials. Beyond adsorption, it investigates into the catalytic capabilities of materials derived from cellulose in CO_2_ conversion, highlighting their function in converting CO_2_ collected into valuable byproducts, including carbonates, methanol, and formic acid. This analysis closes the gap between basic materials research and practical applications in industry by offering a fresh look at carbon capture technologies based on cellulose and outlining the obstacles and opportunities that will be needed to bring them to scale.

## 2. Cellulose Structure and Properties

Cellulose, a cornerstone of plant cell walls, presents an intricate macromolecular structure that underpins its unique physical and chemical properties. Comprising linear chains of glucose molecules linked by β-1,4-glycosidic bonds, cellulose manifests itself as a high-molecular-weight polymer [14]. These linear chains align closely in extended bundles, forming crystalline regions interspersed with less-ordered, amorphous regions. The supramolecular assembly of cellulose imparts it with remarkable strength and rigidity, rendering it a structural scaffold in the cell walls of plants.

### 2.1. Relationship Between Structure and Properties

The intimate relationship between the molecular arrangement of cellulose and its properties is pivotal in understanding its behavior in diverse applications. The crystalline regions, characterized by well-aligned cellulose chains, contribute to the material’s tensile strength and durability. Conversely, the amorphous regions confer flexibility and accessibility, influencing properties such as water absorption and reactivity. Cellulose’s hierarchical structure, from the nanoscale to the macroscale, allows for tailored modifications, providing a versatile platform for materials engineering.

### 2.2. Biodegradability and Sustainability

Cellulose stands as an exemplar of biodegradability, forming a harmonious synergy with the principles of sustainability. The enzymatic degradation of cellulose by microorganisms in the environment facilitates its return to elemental carbon, water, and other benign byproducts. This intrinsic property positions cellulose as a sustainable alternative to synthetic materials with persistent environmental footprints. The biodegradability of cellulose aligns seamlessly with the ethos of green materials design, providing a pathway for the development of eco-friendly solutions in the realm of CO_2_ capture and utilization.

### 2.3. Abundance in Nature and Cost-Effectiveness

A testament to its ubiquity, cellulose is abundantly available in nature, sourced from a myriad of renewable resources, including wood, cotton, and agricultural residues. This abundance not only renders cellulose economically viable, but also diminishes concerns related to resource depletion. The cost-effectiveness of cellulose-based materials becomes a critical factor in the scalability and practicality of CO_2_capture and utilization technologies. The abundance of cellulose offers a sustainable solution that transcends the confines of laboratory research, opening avenues for large-scale implementation in the quest for mitigating carbon emissions.

## 3. CO_2_ Capture Technologies

The imperative to mitigate CO_2_ emissions has propelled the development of diverse capture technologies, each tailored to specific industrial contexts. As a materials scientist and nanotechnologist, the nuanced exploration of these methodologies becomes pivotal in crafting advanced materials that align with the intricacies of CO_2_ capture.

### 3.1. Post-Combustion Capture

Post-combustion capture stands as a prominent strategy deployed to reduce CO_2_ emissions from existing power plants and industrial facilities. This method involves the extraction of CO_2_ from flue gases after the combustion process. Sorbents and solvents play a critical role in this technique, selectively capturing CO_2_ from the gas stream. Advanced materials, including metal-organic frameworks (MOFs) and amine-functionalized polymers, have emerged as promising candidates for enhancing the efficiency and selectivity of post-combustion capture [8]. Their tunable structures and high surface areas offer opportunities for tailoring materials to specific gas separation requirements.

### 3.2. Pre-Combustion Capture

In contrast, pre-combustion capture intervenes at an earlier stage in the energy conversion process, targeting the syngas produced during fuel gasification. This method involves the removal of CO_2_ before combustion occurs, typically utilizing advanced catalysts or absorbents. Nanomaterials, such as supported metal catalysts and nanoporous materials, present avenues for optimizing the efficiency of pre-combustion capture [18,19]. The nanoscale dimensions afford enhanced surface interactions, facilitating the separation of CO_2_ from the gas stream. The design and synthesis of nanomaterials hold promise in overcoming the challenges associated with the energy-intensive nature of pre-combustion capture processes.

### 3.3. Direct Air Capture (DAC) Technologies

An exciting new strategy for reducing greenhouse gas emissions is direct air capture (DAC), which involves capturing CO_2_ straight from the air. The DAC process uses extremely efficient sorbents with good selectivity to function at significantly lower CO_2_ concentrations (~400 ppm), in contrast to traditional carbon capture systems that aim at high-concentration CO_2_ emissions from industrial sources [20]. The standard method for DAC systems involves using solid adsorbents such amine-functionalized materials, metal-organic frameworks, or porous carbon, or liquid sorbents like alkaline solutions, to bind and then release CO_2_ through pressure-swing or heat regeneration.

Combining DAC with CO_2_ sequestration or utilization offers a promising option for reaching zero emissions due to its scalability and flexibility, which allow it to be deployed independently of emission sources [21,22,23]. Furthermore, DAC provides long-term solutions for CO_2_ removal rather than short-term storage options. High energy needs, material deterioration, and cost efficiency are three areas where problems persist. Sorbent regeneration in current DAC techniques consumes a lot of energy, and the cost of recovered CO_2_ per tonne is still much greater than in conventional carbon capture systems [24,25].

Because of their low production costs, malleability in surface chemistry, and ability to be recycled, cellulose-based materials have recently been investigated forpossible DAC uses. Research on cellulose-calcium hybrid systems, for example, has shown that, under moderate circumstances, it is possible to effectively absorb CO_2_ from the air, pointing to a potential approach towards DAC systems that are both more sustainable and economically feasible [26]. The development of DACs based on cellulose is still in its infancy, but there is continuous effort to improve these materials adsorption efficiency, stability, and compatibility with current DAC systems. 

To improve economic feasibility and enable large-scale deployment, the development of low-energy, sustainable sorbents will be important as DAC technologies continue to mature. An intriguing area of research is the incorporation of cellulose-based materials into DAC systems. This might lead to a decrease in the use of synthetic sorbents, which are energy intensive, and the promotion of solutions that do not produce any carbon emissions.

### 3.4. Industrial Applications and Challenges

The deployment of CO_2_ capture technologies in industrial settings spans a spectrum of sectors, including power generation, cement production, and chemical manufacturing. Each application, however, presents unique challenges that demand innovative solutions. Materials scientists grapple with the optimization of sorbents, catalysts, and membranes to withstand harsh operating conditions, ensuring longevity and cost-effectiveness. The integration of nanomaterials, with their tailored properties and enhanced reactivity, holds the potential to address these challenges [27,28]. Challenges such as material stability, regeneration efficiency, and scalability necessitate a multidisciplinary approach, encompassing materials design, engineering, and process optimization.

## 4. Need for Sustainable Adsorbents

Traditional adsorbents, such as activated carbons and zeolites, have been stalwarts in CO_2_ capture methodologies. However, the environmental ramifications of their production and deployment underscore the urgency for more sustainable alternatives. The manufacture of activated carbon, for instance, necessitates high-temperature carbonization processes, contributing substantially to energy consumption and carbon emissions during production [3,29]. This energy-intensive production phase raises concerns about the overall carbon footprint of traditional adsorbents.

Furthermore, the raw materials for these adsorbents are often derived from non-renewable sources, leading to increased pressure on natural resources. The extraction and processing of these materials contribute to ecosystem disruption and environmental degradation. Additionally, the regeneration of traditional adsorbents frequently involves energy-intensive procedures, amplifying the ecological impact of these widely used materials [11,30,31]. Recognizing these environmental challenges, the quest for sustainable adsorbents becomes imperative in mitigating the ecological toll of CO_2_ capture technologies.

### Advantages of Cellulose-Based Materials

Cellulose, as the principal structural component of plant cell walls, offers a compelling alternative to traditional adsorbents, with intrinsic advantages rooted in sustainability. Derived from renewable biomass sources, including wood, cotton, and agricultural residues, cellulose addresses concerns related to resource depletion and non-renewable feedstocks [14,32,33,34,35]. The utilization of cellulose as a precursor for adsorbents aligns seamlessly with the principles of green chemistry, emphasizing the importance of renewable and environmentally benign materials.

The distinctive properties of cellulose, including its high surface area and tunable chemistry, present an opportunity for tailored modifications at the nanoscale. Leveraging nanotechnological approaches allows for the design of cellulose-based adsorbents with enhanced CO_2_ adsorption capacities and selectivities [34,36]. The renewable nature of cellulose also extends to its end-of-life disposal, with cellulose-based adsorbents exhibiting biodegradability, aligning with the ethos of a circular and sustainable economy.

Beyond their environmental benefits, cellulose-based materials are quite appealing for major CO_2_ capture applications because of their sustainability, cost-effectiveness, and industrial suitability. From a sustainability standpoint, cellulose comes from plentiful, renewable biomass sources, so lessening reliance on fossil-based adsorbents and lessening of the environmental impact of material manufacture. Unlike synthetic substitutes that call for energy-intensive manufacturing and produce harmful consequences, cellulose-based adsorbents fit green chemistry ideas, therefore supporting a circular carbon economy.

Economically speaking, cellulose-based materials offer a somewhat affordable substitute for conventional adsorbents, including activated carbon, metal-organic frameworks (MOFs), and amine-based sorbents, which can involve high synthesis costs and difficult regeneration techniques. Cellulose’s availability from industrial waste streams, forestry byproducts, and agricultural leftovers reduces material costs even further, thereby making large-scale deployment more practical. Furthermore, the manufacturing methods for cellulose-based adsorbents—such as nanostructuring and chemical functionalization—can be tuned to improve CO_2_ adsorption efficiency without appreciably raising manufacturing costs.

Another main benefit of cellulose-based materials is industrial feasibility since they provide structural flexibility, chemical adaptability, and compatibility with current carbon capture technologies. Under mild conditions, cellulose-based adsorbents show great reusability and regeneration potential, unlike conventional materials that could suffer from stability problems or need expensive regeneration procedures. From post-combustion capture in power plants to direct air capture systems, their capacity to be processed into films, aerogels, and composite adsorbents helps them to be integrated into many CO_2_ capture technologies. Moreover, developments in scalable modification methods, including composite hybridization and amine functionalization, have improved the adsorption effectiveness and selectivity of cellulose-based materials, therefore enabling their competitiveness with conventional capture systems. Cellulose-based adsorbents bridge the gap between laboratory research and practical uses by tackling sustainability, cost-effectiveness, and industrial practicality, therefore offering a viable road towards scalable and environmentally responsible CO_2_ capture systems.

Beyond material properties, the fabrication processes for cellulose-based adsorbents can be tailored to enhance their environmental profile. Techniques such as solvent-free fabrication and the use of green solvents contribute to minimizing the ecological impact of production [33,35,37,38,39]. The integration of cellulose-based materials in CO_2_ capture technologies not only addresses environmental concerns but also propels the field towards a more sustainable and ethical trajectory.

## 5. Design and Synthesis of Cellulose-Based Adsorbents

### 5.1. Chemical Functionalization

At the molecular frontier of cellulose modification lies the artful strategy of chemical functionalization. This approach involves the deliberate alteration of cellulose chemical composition through the introduction of functional groups, imparting novel physicochemical properties to the material. Cellulose derivatives, such as cellulose acetate and carboxymethyl cellulose (CMC) have gained prominence in the realm of CO_2_ capture due to their tunable surface reactivity and solubility [17,34,37,40,41,42]. Chemical functionalization not only tailors the adsorption sites on cellulose but also influences the overall hydrophilicity and affinity towards CO_2_ molecules.

Advanced methodologies, including graft copolymerization and esterification reactions, enable precise control over the type and density of functional groups introduced onto the cellulose backbone. This level of precision allows materials scientists to design cellulose-based adsorbents with tailored affinities for CO_2_, presenting a promising avenue for optimizing the efficiency of the capture process [43,44,45,46]. The judicious choice of chemical modifiers and reaction conditions becomes pivotal in sculpting cellulose-based adsorbents with enhanced selectivity and capacity.

### 5.2. Modifications for Enhanced Performance

Beyond the realm of chemical functionalization, structural modifications stand as a cornerstone for elevating the performance of cellulose-based adsorbents. The inherent hierarchical structure of cellulose, comprising crystalline and amorphous regions, offers a canvas for precision engineering. Nanotechnology plays a pivotal role in this domain, allowing materials scientists to tailor cellulose at the nanoscale for optimized CO_2_ adsorption.

Nanocellulose, encompassing nanofibers, nanocrystals, and bacterial cellulose, emerges as a versatile material for structural modifications. The high surface area-to-volume ratio of nanocellulose facilitates increased interaction sites for CO_2_ molecules [38,41,47]. Furthermore, the introduction of nanoscale porosity through techniques like templating or aerogel formation enhances the accessibility of reactive sites, fostering a higher degree of CO_2_ capture efficiency [48]. The synthesis of nanocomposites by incorporating nanoparticles, such as graphene oxide or metal-organic frameworks (MOFs), into cellulose matrices further amplifies the adsorption capabilities, showcasing the synergy between nanotechnology and cellulose design [32].

The strategic combination of chemical functionalization and structural modifications not only amplifies the inherent properties of cellulose-based adsorbents, but also extends their applicability to diverse environmental conditions. This multidimensional approach underscores the evolution of cellulose from a mere structural biopolymer to a sophisticated and adaptable material for CO_2_ capture technologies.

## 6. Synthesis Methods

The synthesis of cellulose-based adsorbents traverses a diverse array of techniques, each tailored to harness the unique properties of cellulose for optimal CO_2_ capture. Among the prominent methods is the solution-based fabrication approach, where cellulose is dissolved in suitable solvents, allowing for precise control over the materials morphology and structure. Techniques such as regeneration and electrospinning enable the formation of cellulose fibers, films, or nanofibers with enhanced surface areas for improved CO_2_ adsorption [49].

Nanotechnological methods play a pivotal role in refining cellulose-based adsorbents. The bottom-up assembly of nanocellulose, including nanocrystals or nanofibrils, allows for the creation of materials with well-defined nanostructures. Templating approaches, such as using sacrificial templates or self-assembly processes, facilitate the generation of porous cellulose structures, maximizing the availability of active sites for CO_2_ adsorption [3,50,51]. Additionally, advanced techniques like spray drying and freeze-drying contribute to the formation of aerogels or porous structures, augmenting the overall performance of cellulose-based adsorbents [36,52,53].

Solid-state methods, including ball milling and mechanical milling, offer alternatives for the synthesis of cellulose-based adsorbents. These techniques induce structural changes in cellulose, generating amorphous regions and increasing surface reactivity. The resulting materials exhibit enhanced adsorption capacities due to their altered crystalline structures and increased accessibility of active sites [36].

### Incorporation of Additives and Modifiers

The optimization of cellulose-based adsorbents often extends beyond the intrinsic properties of cellulose, involving the strategic incorporation of additives and modifiers to fine-tune performance. Additives, such as nanoparticles or functionalized polymers, are integrated to impart additional functionalities to the adsorbent matrix. For instance, the incorporation of metal-organic frameworks (MOFs) or graphene oxide nanosheets enhances the adsorption efficiency by introducing additional binding sites for CO_2_ molecules [10,32,44,54,55].

Modifiers are introduced to tailor the surface chemistry of cellulose-based adsorbents. Chemical functionalization with amino groups or other reactive moieties enhances the adsorbent’s affinity towards CO_2_, providing a more selective and efficient capture process [43,45,46,56,57,58]. The judicious selection of modifiers enables control over factors such as hydrophobicity, which can impact the adsorption performance in humid conditions. Furthermore, the integration of environmentally benign modifiers aligns with the overarching goal of sustainability in materials design.

The synergy between cellulose, additives, and modifiers presents a canvas for precise control over the physicochemical properties of the adsorbent. The incorporation of these elements serves as a testament to the sophistication of modern materials science, where synergistic interactions are engineered to address the complex challenges associated with CO_2_ capture.

In synthesizing cellulose-based adsorbents, the amalgamation of diverse fabrication techniques and the strategic incorporation of additives and modifiers underline the multidimensional nature of materials design. This advanced approach not only pushes the boundaries of innovation but also positions cellulose-based materials as frontrunners in the quest for sustainable and efficient solutions in CO_2_ capture.

## 7. Surface Modification for Selective CO_2_ Capture

### 7.1. Tailoring Surface Properties for Improved Adsorption

The surface of cellulose-based adsorbents serves as the frontline interface for interacting with CO_2_ molecules, and as such, tailoring its properties becomes paramount. Surface engineering approaches encompass a spectrum of strategies aimed at optimizing parameters such as surface area, porosity, and surface chemistry. Nanocellulose, with its inherently high surface area, offers a versatile platform for surface modifications [37,39,41,42,43,59,60].

The introduction of nanoporosity through controlled templating or aerogel formation amplifies the active surface sites available for CO_2_ adsorption. This nanoscale tailoring enhances the kinetics of the adsorption process, ensuring rapid and efficient capture of CO_2_ molecules [19,60,61]. Furthermore, the augmentation of surface roughness through methods like ball milling or surface etching contributes to increased surface interactions, optimizing the utilization of available cellulose for adsorption purposes.

Functional groups play an important role in surface property tailoring. The controlled incorporation of specific functional groups, such as amino or hydroxyl groups, onto the cellulose surface improves the adsorption affinity for CO_2_. This nuanced engineering of surface chemistry ensures that the adsorbent selectively interacts with CO_2_ molecules, minimizing the potential interference from other gases present in the environment [43,45]. The integration of these surface engineering approaches synergistically refines the cellulose-based adsorbent, transforming it into a highly effective and selective tool for CO_2_ capture.

### 7.2. Functionalization Methods and Their Impact on Selectivity

The art of functionalization, a cornerstone in surface modification, which imparts tailored functionalities onto cellulose-based adsorbents. Various methods, including wet chemistry and gas-phase reactions, offer precise control over the introduction of functional groups. Wet chemistry methods, such as grafting reactions and esterification processes, enable the covalent attachment of functional moieties onto the cellulose surface.

The selection of functional groups dictates the adsorbents specificity towards CO_2_. For instance, amine functionalization has been widely employed due to the Lewis acid-base interaction between amines and CO_2_. The amine groups act as anchor sites for CO_2_ molecules, enhancing the overall adsorption capacity and selectivity [44]. The judicious choice of functionalization methods ensures the creation of tailored adsorbents that exhibit high specificity for CO_2_, even in the presence of competing gases.

Gas-phase functionalization techniques, such as chemical vapor deposition (CVD) or plasma treatment, offer advantages in terms of scalability and uniformity. These methods enable the controlled introduction of functional groups without altering the bulk properties of cellulose. Plasma treatment, for example, imparts a high degree of surface reactivity, allowing for selective functionalization and improved CO_2_ adsorption performance [62].

The impact of functionalization methods on selectivity extends beyond the immediate adsorption process. It influences the regeneration efficiency of the adsorbent, determining its cyclic stability and long-term efficacy. The interplay between surface functionalization and selectivity thus encapsulates the intricate dance between chemical precision and environmental application.

## 8. Advances in Controlling Surface Chemistry

Effects on CO_2_ Capture Efficiency: The surface chemistry of cellulose-based adsorbents plays a pivotal role in dictating their interaction with CO_2_ molecules. Recent advances in controlling surface chemistry aim at enhancing the adsorption efficiency by tailoring the material’s surface to selectively attract and capture CO_2_. The strategic introduction of functional groups, such as amines or hydroxyls, imparts unique chemical moieties on the cellulose surface, facilitating strong and specific interactions with CO_2_.

These surface modifications, often achieved through sophisticated chemical techniques, influence the kinetics and thermodynamics of CO_2_ adsorption. The presence of amine groups, for example, enables Lewis acid–base interactions with CO_2_, leading to higher adsorption capacities and improved selectivity [63,64,65]. The controlled adjustment of surface chemistry also affects the adsorption–desorption equilibrium, enhancing the adsorption process total efficacy. Moreover, advances in nanoparticle integration and nanoscale surface engineering have opened avenues for tailoring the surface morphology of cellulose-based adsorbents. The introduction of nanostructures or nanopores amplifies the available surface area, providing more active sites for CO_2_ adsorption. Nanoscale modifications not only enhance the adsorption capacity but also contribute to rapid kinetics, making the process more efficient and responsive to fluctuating CO_2_ concentrations.

The success stories of surface-modified cellulose materials underscore the transformative impact of controlled surface chemistry on CO_2_ capture. One notable case involves the use of amine-functionalized cellulose nanofibers. Through a combination of wet chemistry techniques, amine groups were introduced onto the cellulose surface. The resulting material exhibited a significant increase in CO_2_ adsorption capacity due to the enhanced reactivity of amine functionalities [60].

Another compelling case study involves the integration of metal-organic frameworks (MOFs) with cellulose. The surface modification with MOFs not only expanded the available surface area but also introduced tailored binding sites for CO_2_ molecules. The synergistic combination of cellulose’s structural integrity and the tunable porosity of MOFs resulted in a highly efficient adsorbent with enhanced selectivity for CO_2_ over other gases.

Furthermore, the advent of plasma treatment as a surface modification technique showcased promising results. Cellulose aerogels treated with plasma exhibited improved surface reactivity, leading to enhanced CO_2_ adsorption performance. The non-thermal nature of plasma treatment allows for precise control over the introduction of functional groups, demonstrating a powerful approach in tailoring surface chemistry for specific gas adsorption applications [9,66,67,68,69,70,71]. These case studies exemplify the success of surface-modified cellulose materials in achieving superior CO_2_ capture performance. The intricate interplay between surface chemistry and material properties showcased in these examples highlights the strides made in tailoring cellulose-based adsorbents for optimal performance in real-world environmental applications.

## 9. Applications of Cellulose-Based Materials in CO_2_ Capture

The term “carbon capture” refers to the method of removing carbon dioxide gas from combustion-based sources like power plants and industrial facilities. Chemical absorption, physical absorption, solid adsorption, and membrane separation are various carbon capture exiting techniques. One of the more established approaches is chemical absorption, which involves removing CO_2_ from combustion sources using certain chemicals and then reusing and recycling the chemical absorbent in order to produce CO_2_ with a high level of purity. Films, aerogels, and solid adsorbents are the three main materials that CO_2_ adsorbents can take. Activated carbon, silica, alumina, MOFs, and porous polymers are commonly utilized as carriers and pore-making mediums in carbon capture [30,31,54]. Activated carbon is effective at removing gases from the environment, but it is expensive, doesn’t last long, and isn’t sensitive to some gases [72]. The stability, affordability, and selectivity of MOF are questionable, according to recent studies [10,73]. According to Ye et al. (2024), porous polymers made of silicon dioxide and alumina have a poor ability to absorb substances [74]. Additionally, cellulose-based materials have numerous benefits, such as being easily modifiable, having a large range of sources, being renewable, and having good adsorption selectivity. To improve the adsorption performance and durability of cellulose-based materials, one can manipulate their pore structure and chemical characteristics by adjusting the pressure, temperature, and chemical treatment [14,40,60,75].

### 9.1. Composite Film

**Figure 1:**
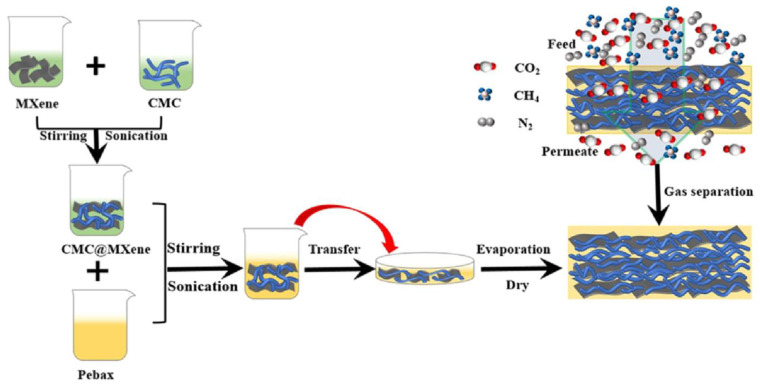
Process flow diagram for the fabrication and gas transfer of Pebax/CMC@MXene MMMs (Adapted with permission from ACS [76]).

Combining 2D-MXene nanoplatelets with branched CMC, Luo et al. (2022) [76] created a high-performance self-supporting mixed matrix membrane for CO_2_ gas separation in flue gas and natural gas (Figure 1).

**Figure 2:**
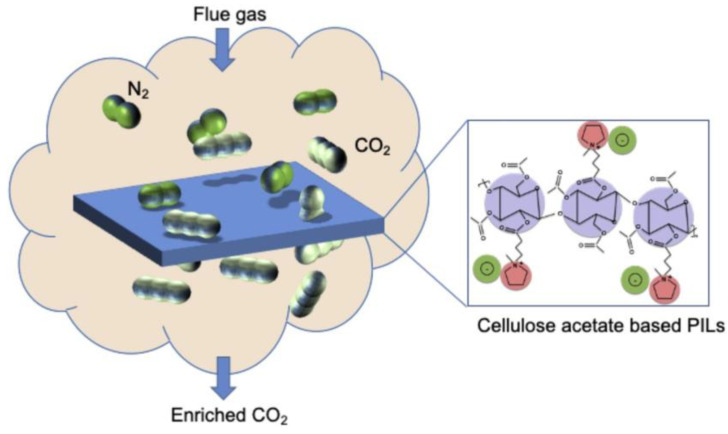
The process of CO_2_/N_2_ penetration into a film based on cellulose acetate (Adapted with permission from Elsevier [77]).

The independent Pebax/CMC@MXene MMMs that were constructed demonstrated highly selective CO_2_/N_2_ and CO_2_/CH_4_ gas mixture separation capabilities, as well as good CO_2_ permeability. The CO_2_ permeability and CO_2_/N_2_ selectivity of Pebax/CMC/@MXene MMMs were 521 GPU and 40.1, respectively, at 1.5 mg/mL of MXene nanosheets. To enhance the separation capabilities of cellulose acetate (CA), Nikolaeva et al. (2021) [77] attached ionic liquid-like suspending agents (1-methylimidazole, 1-methylpyridine, and 2-hydroxyethyl dimethylamine, or HEDMA) to a CA backbone (Figure 2).

The polymers were coated using a semi-automatic technique to create thin film composite (TFC) membranes. As the HEDMA content increased, the CO_2_ and N_2_ permeability decreased in the CO_2_/N_2_ mixed gas permeation studies, and the selectivity for CO_2_/N_2_ decreased at first, but then gradually increased.

### 9.2. Composite Aerogel

**Figure 3:**
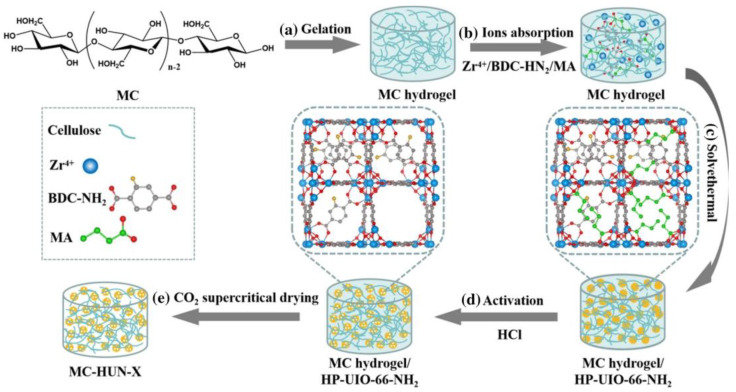
Schematic of steps involved in making MC-HUN aerogel (**a**) Sol–gel transformation of MC, (**b**) Adsorption of metal ions into the MC hydrogel, (**c**) Growth of HUN-X within MC gel networks via a solvent-thermal reaction, (**d**) Activation of HUN-X using HCl treatment, (**e**) Final MC-HUN-X aerogel obtained through supercritical CO_2_ drying. (Adapted with permission from [52]).

A series of hybrid aerogels were produced by combining cellulose aerogels with MOFs, which are large-pore hierarchical porous metal-organic frameworks [52]. The mixture was then modified with monocarboxylic acid (MA) and in situ grown HP-UIO-66-NH_2_ was added to the mixture. In Figure 3 MC-HUN-X, X is the carbon atom count. Changing the MA chain length allows one to adjust the pore size of HP-MOFs. The maximum CO_2_ adsorption capacity (1.90 mmol/g at 298 K and 1 bar) and adsorption selectivity (13.02 for CO_2_/N_2_ and 2.40 for CO_2_/CH_4_, respectively) were achieved by MC-HUN with medium pore size when the MA carbon number was 4.

**Figure 4:**
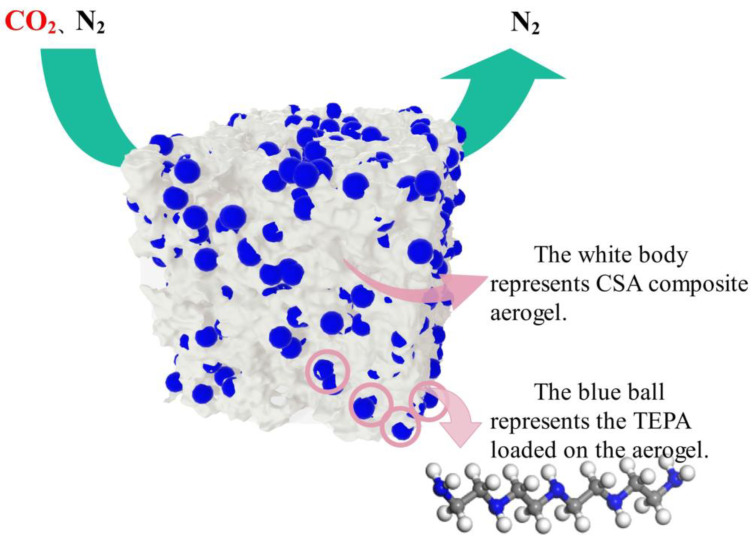
Diagram representing TEPA loaded silicon aerogel. (Adapted with permission from [78]).

A new class of metal-organic frameworks (MOFs) with a zeolite topology, called zeolite imidazolate frameworks (ZIFs), is gaining attention as a potential material for storing carbon dioxide. Using skimmed cotton and the sol-gel technique, Zhou et al. (2021) created cellulose whisker/silica composite aerogels from TEOS and an alkaline silica solution as the precursor to the silica [78]. In Figure 4, we can see the silica aerogel that has been treated using tetraethylenepentamine (TEPA). With a maximum adsorption capacity of 2.25 mmol/g at 70% TEPA loading, the CSA-TEPA 70% aerogel demonstrated outstanding adsorption performance. The use of this adsorbent in the capture of carbon dioxide is highly encouraging. Many people are interested in studying MOFs because of their tunable microporosity, structural variety, large surface area, strong affinity for CO_2_, and adjustable structure.

**Figure 5:**
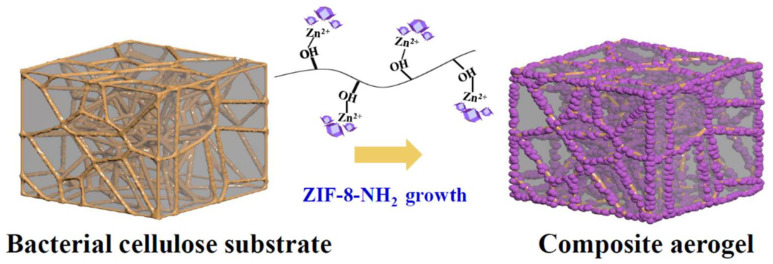
Schematic depicting the production process of ZIF-8-NH2@BC foam (Adapted with permission from [44]).

Using bacterial cellulose (BC) as a substrate, Ma et al. (2021) [44] created composite aerogels of aminofunctionalized ZIF-8 (ZIF-8-NH2) as shown in Figure 5.

**Figure 6:**
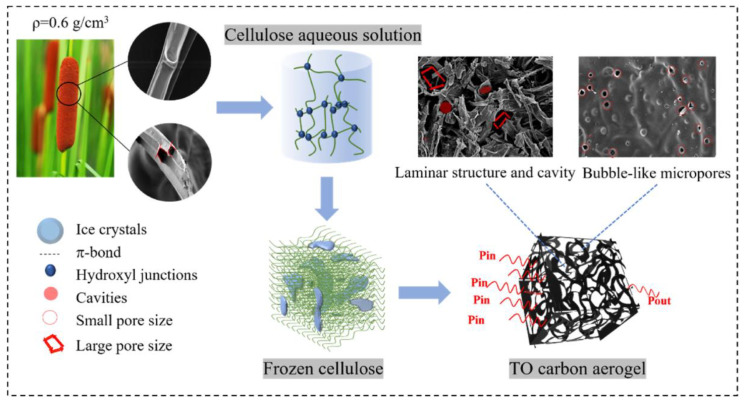
Cellulose structure and shape, and the process of creating Typha Orientalis (TO) carbon aerogel. (Adapted with permission from [79]).

High interfacial affinity and compatibility were achieved in composites made from ZIF crystals that were uniformly encased around cellulose fibers. This was achieved through hydroxyl group-zinc ion chelation. With a capacity of 1.63 mmol/g, the aerogel is highly effective at adsorbing CO_2_. Cellulose has oxygen and hydroxyl groups that zinc ions can combine with to create complexes. Without the use of a binder, ZIF-8-NH_2_ crystals formed the linker and encased the BC chains when 2-methylimidazole and 2-aminobenzimidazole were added. Modifying the concentration of the organic linker allowed for the optimization of the ZIF-8 amino group loading. By acid-treating Typha Orientalis (TO) cellulose and then effectively freeze-casting and freeze-drying it, Cheng et al. (2023) [79] created cellulose carbon aerogel (CA) with a three-dimensional network structure, as shown in Figure 6.

They then activated CA with KOH to improve its energy storage capabilities. The material could adsorb 16 mmol/g CO_2_, 123.31 mg/g oxylene, and 124.57 mg/g o-dichlorobenzene, and it could store 0.6 wt.% hydrogen at room temperature. As a support, An et al. (2023) [80] used cellulose-based porous carbon aerogel (CPCa) to graft polyethyleneimine (PEI). To maximize the synergistic impact and enable efficient separation of CO_2_ from industrial flue gas, the PEI-grafted cellulose-based porous carbon aerogel (PEI-CPCa) was designed with a rationalized molecular arrangement and its pore effect. Using CO_2_ and N_2_ as simulated gases, PEI-CPCa was able to adsorb 5.58 mmol/g of CO_2_ with a separation factor of 113.1.

### 9.3. Composite Solid Adsorbents

**Figure 7:**
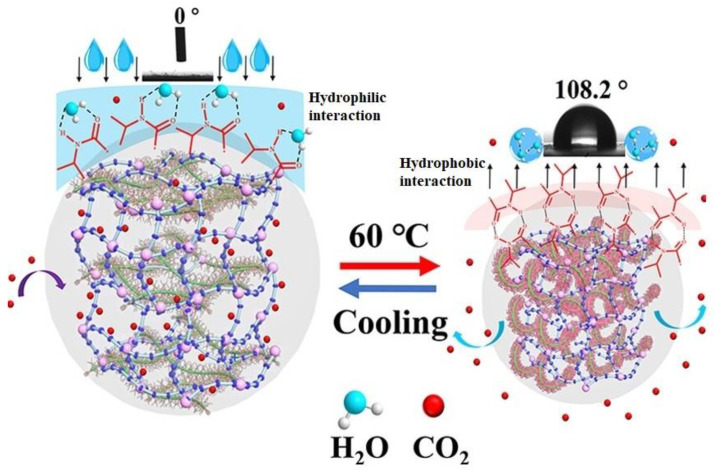
Mechanism of TRCNF/PEIA low-temperature regeneration. Shrinkage above the LCST promotes CO_2_ desorption, surface hydrophobicity (WCA = 106.4°) aids water removal, and hydrogen bonding stabilizes carbamate, preventing urea formation. This enables efficient CO_2_ desorption at 60 °C. (Adapted with permission from [83]).

The use of solid adsorbent materials in CO_2_ capture and concentration yields excellent results since these materials are safe, environmentally friendly, and have low adsorption energy consumption. They are also weakly corrosive. Both low-temperature and high-temperature adsorbents are solid adsorbents. Calcium oxide adsorbent material is one example of a high-temperature adsorbent material; it can remove carbon dioxide even when heated to 600 °C. By combining the acidic gas carbon dioxide with the alkaline calcium oxide adsorbing material, a chemical process is initiated, resulting in the formation of calcium carbonate. Activated carbon and zeolite are examples of materials that may absorb substances at low temperatures. As an example, consider zeolite, a naturally occurring silica-aluminate that serves as a sieve for molecules, an adsorbent, a medium for ion exchange, and a catalyst. Its pores are uniformly nanometer-scale, creating interconnecting channels and a mesh structure. Chen et al. (2021) [81] developed a two-step palletization process for the preparation of in-shell CaO-based adsorbent microspheres. Extrusion spheronization was used to create cores with a high activity level; these cores comprised 80% calcium hydroxide and 20% cellulose. As a means of creating pore-forming templates, CaO shells were coated with varying concentrations of cellulose, ranging from 0% to 40% by weight. The porosities within the shell can be varied by adding varying amounts of cellulose to it. The most effective CO_2_ absorbents are cellulose-templated pellets manufactured from calcium oxide with an outer layer consisting of 40% cellulose. In particular, the CO_2_ collection capability of these pellets is 0.144 g/g. Better CO_2_ capture performance is achieved as a result of the porous shells drawing CO_2_ closer to the very active core pellets. Thermoresponsive bionic fiber adsorbents based on solar-triggered regenerating cellulose nanofibers (CNF) were developed by Lu et al., 2022 [82]. The substrate backbone of CNF-TBFA is cellulose nanofiber (CNF), and in thermosensitive polymers, the amino groups of poly(niisopropylacrylamide) (PNIPAm), graphene oxide nanoparticles (GO), and polyethyleneimine (PEI) serve as the thermal response switch, photothermal conversion switch, and CO_2_ adsorption site, respectively. The CNF-TBFA that was made showed a low photo-triggered regeneration temperature and a high CO_2_ adsorption capacity when water was present. Over the course of ten adsorption–desorption cycles, the adsorbents CO_2_ capture capability remained constant. Renewable solar energy can be used to regenerate the adsorbent, which has a high CO_2_ adsorption capacity of 6.52 mmol/g. The findings provide hope for the future of activated carbon with microcrystalline cellulose for CO_2_ capture. Thermosensitive cellulose nanofibers (TRCNF) were rapidly crosslinked with epichlorohydrin to create a new solid amine adsorbent (TRCNF/PEIA) by Lin et al. (2021) [83]. This adsorbent has a high amine density and a high content of thermosensitive smart groups. The second one was made by using Ce (IV)-initiated radical polymerization to graft n-isopropylacrylamide (NIPAm) onto cellulose nanofibers (CNF). While tested in water at 25 °C, the TRCNF/PEIA that was produced showed an impressively high adsorption capacity of 6.74 mmol/g. Figure 7 provides a mechanism of the low-temperature regeneration mechanism of TRCNF/PEIA. (1) The adsorbent contracts when the temperature rises above the LCST, which aids in the desorption of carbon dioxide; (2) TRCNF/PEIA surfaces acquire hydrophobic properties (WCA = 106.4°) as a result of its thermo-responsive properties; (3) the hydroxyl group on the surface of the thermal nanofiber can interact with amino carbamate or carbamate through hydrogen bonding, stabilizing the formed carbamate and inhibiting the formation of urea; (4) the desorption temperature is effectively lowered.

**Figure 8:**
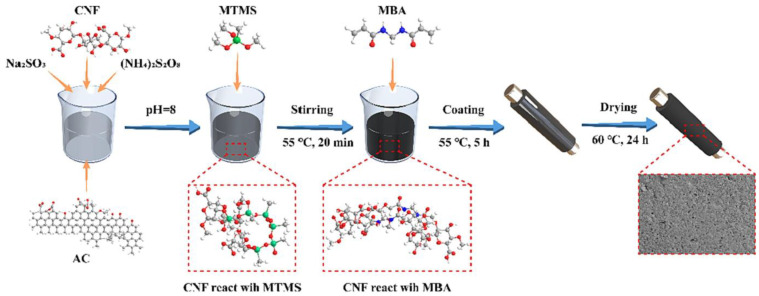
Schematic of the hydrophobic coating preparation process. CNF dispersion (6 wt%) was mixed with AC and stirred, followed by the addition of initiators and pH adjustment. Various amounts of MTMS were incorporated, and the mixture was heated with MBA. The resulting slurry was coated on a metal tube and dried at 60 °C for 24 h to form the hydrophobic coating. (Adapted with permission from [84]).

Also, to improve activated carbon adsorption capability, Yu et al. (2023) suggested coating the outside of a metal tube with a hydrophobic carbon layer as a quick CO_2_ adsorption/desorption method [84]. To create the covers, activated carbon from coconuts were used as a support, carbonylated cellulose nanofibers (CNF) as a binder, and methyltrimethoxysilane as a hydrophobic modifier. Figure 8 shows that groups containing oxygen enhance the surface polarity and adsorption of tiny, weakly polar molecules like carbon dioxide. The original activated carbon had an equilibrium adsorption capacity of 34.3% for wet flue gas (15 vol% CO_2_ + 4.7 vol% H_2_O) and 39.4% for dry flue gas (15 vol% CO_2_) at 40 °C and 1.0 bar, respectively. SAC-6-1.0 achieved an efficiency that was 382.4% higher in the former case and 34.5% higher in the latter. More than 97% of its initial adsorption capacity remained after 10 reuse cycles.

The CO_2_ adsorption capability of the porous metal oxide supports on K_2_CO_3_-based adsorbent pellets was investigated by Zheng et al. (2023) by including several porous supports (TiO_2_, ZrO_2_, and SiO_2_) into graphite-cast K_2_CO_3_ adsorbent particles [85]. The CO_2_ adsorption performance of ZrO_2_-supported potassium carbonate adsorbent microspheres was approximately 0.93 mmol/g, which had the best performance compared to TiO_2_- or SiO_2_-supported microspheres. High temperature calcination can cause two things: first, particles can clump together and grow, which can ruin the microstructure; second, potassium carbonate and silica can react to create inactive eutectic compounds. Preventing particle collapse and surface graphite layer loss during calcination of SiO_2_-supported potassium carbonate adsorbent beads requires reducing the temperature to below 500 °C. There was also an increase in CO_2_ adsorption to 1.30 mmol/g when 20 wt% microcrystalline cellulose was added to the SiO_2_-loaded K_2_CO_3_ adsorbent pellets.

### 9.4. Technology Readiness Level (TRL) of Cellulose-Based Adsorbents

From basic research (TRL 1) to full-scale commercial use (TRL 9), the Technology Readiness Level (TRL) is a generally agreed upon framework for evaluating the development stage of technologies. Currently operating in TRL 7–9, conventional CO_2_ capture materials including amine-based adsorbents, activated carbon, zeolites, and metal-organic frameworks (MOFs) have been extensively investigated and applied in industrial-scale carbon capture systems [10,32,44,54,55,63,64,65]. These materials do, however, have major disadvantages that compromise their large-scale economic viability: high regeneration energy requirements, low stability, and high manufacturing costs.

By comparison, depending on the particular modification and use, cellulose-based adsorbents are now at TRL 3–6. Mostly tested at the laboratory scale (TRL 3–4), unmodified cellulose materials including films and cellulose aerogels show good adsorption capabilities but need further optimization for industrial use [52]. With pilot-scale studies assessing their capability for CO_2_ capture in simulated industrial conditions, advanced chemically modified cellulose adsorbents, including amine-functionalized cellulose and hybrid nanocellulose composites, have proceeded to TRL 5–6. More study is needed, though, to improve adsorption efficiency, regeneration stability, and cost-effectiveness for broad application.

With continuous pilot studies looking at MOF-based adsorbents for post-combustion CO_2_ capture, activated carbon and zeolites are extensively used in industrial applications including power plants, chemical refineries, and gas separation units. Though they are mostly used in prototype carbon capture devices, laboratory research, and developing direct air capture systems [30], cellulose-based materials are still in their early phases of industrial application. Further studies on long-term adsorption stability, scalability of synthesis, and real-world performance under flue gas conditions will help to translate research into commercial feasibility.

The cost-effectiveness and sustainability of cellulose-based adsorbents over traditional materials are main benefits. With readily available and renewable raw materials—wood pulp, agricultural wastes, and bacterial cellulose—cellulose-based adsorbents have much reduced material costs. Comparatively to $100–500 per kg for MOFs and $50–200 per kg for high-grade activated carbon the projected production cost for cellulose aerogels varies from $10–50 per kg [72]. Advanced surface functionalization, such as amine grafting or hybridization with nanoparticles, raises the cost, sofurther optimization is required to balance performance with cost.

## 10. Cellulose Composites in Carbon Conversion

The conversion of CO_2_ into chemicals and fuels represents a pivotal strategy in addressing environmental challenges while harnessing renewable resources. This process, known as carbon capture and utilization (CCU) or carbon capture and conversion (CCC), involves the transformation of CO_2_, a greenhouse gas linked to climate change, into valuable chemical compounds and sustainable fuels. There are various methods that can accomplish this, such as biological, chemical, electrochemical and photochemical reactions. Chemical reactions are the most typical way to convert CO_2_. CO_2_ combines with other chemicals to create fuels or other useful compounds. Mineralization, fuel, chemical, and construction material production all make use of CO_2_. The chemical industry and energy production both make extensive use of CO_2_ conversion, which has four primary benefits, one of which is the potential to lessen reliance on fossil fuels: (1) Converting water into hydrocarbons; (2) oxidizing CO_2_ to produce gaseous hydrocarbons; (3) cycloaddition to polymers; and (4) reducing CO_2_ to carbon monoxide. Activation is challenging, though, because carbon dioxide with linear chemical bonds is a chemically inert molecule. Because of this, understanding the rate of CO_2_ conversion is quite challenging. As a result, improving the efficiency of CO_2_ conversion frequently requires the introduction of various catalytic metals, which significantly hinders the development of carbon utilization technologies. Cellulose and cellulose derivatives offer a novel approach to carbon utilization due to its commercial manufacture and three-dimensional open network structure.

### 10.1. Carbonate Salts

**Figure 9:**
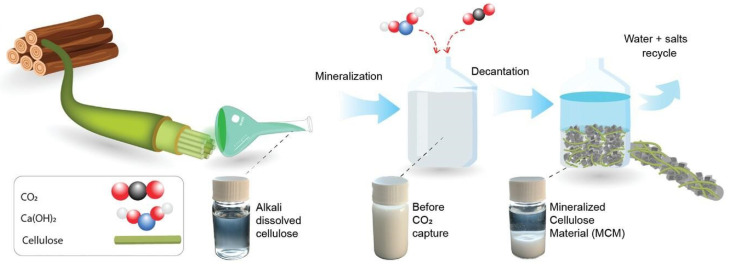
Schematic representation of mineralization process (Adapted with permission from [86]).

The formation of carbonate salts from carbon dioxide involves a chemical process known as carbonation. Carbonation is the reaction of CO_2_ with metal oxides, hydroxides, or other alkaline substances to produce carbonate salts. This process is particularly significant in carbon capture and storage technologies as it provides a means to sequester carbon dioxide from industrial emissions. One common form of carbonation involves the reaction of CO_2_ with metal oxides or hydroxides to form metal carbonates. For example, calcium oxide or calcium hydroxide can react with CO_2_ to produce calcium carbonate. In order to speed up the process of CO_2_ conversion, cellulose can be utilized as a catalyst. Figure 9 shows the organic–inorganic viscous system that Reyes et al. (2023) created by mineralizing cellulose minerals (MCM) [86].

**Figure 10:**
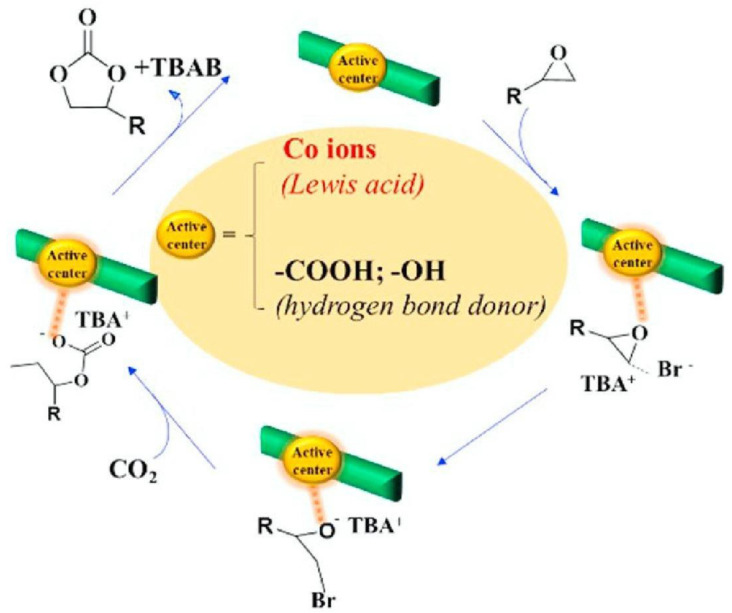
Mechanism diagram showing the steps involved in the catalytic cycloaddition of epoxides and carbon dioxide to produce cyclic carbonates (Adapted with permission from [42]).

Ceramic glazes and cementitious composites can benefit from the mineralized cellulose’s ability to absorb carbon dioxide and transform it into calcium carbonate. Simultaneously, a stone-like structure made of the cellulose-rich gel can be extruded and evaluated as a synthetic coral stone for reef regeneration. Hu et al. (2021) achieved a stable heterogeneous catalyst by partially immobilizing Co(III) on CNC surfaces [42]. This catalyst is capable of catalyzing the cycloaddition process of carbon dioxide at mild circumstances, allowing for the synthesis of cyclic carbonates. In particular, the epoxide ring is opened by nucleophilic attack on the tetrabutylammonium bromide bridge. The next step in opening the epoxide ring is for a Lewis acid center or a hydrogen bond donor to step in. The subsequent insertion of carbon dioxide occurs as a result of the reaction between the negatively charged oxygen atom and CO_2_, which is electrophilic (Figure 10). Finally, the cyclization step yields the cyclic carbonate ester product.

**Figure 11:**
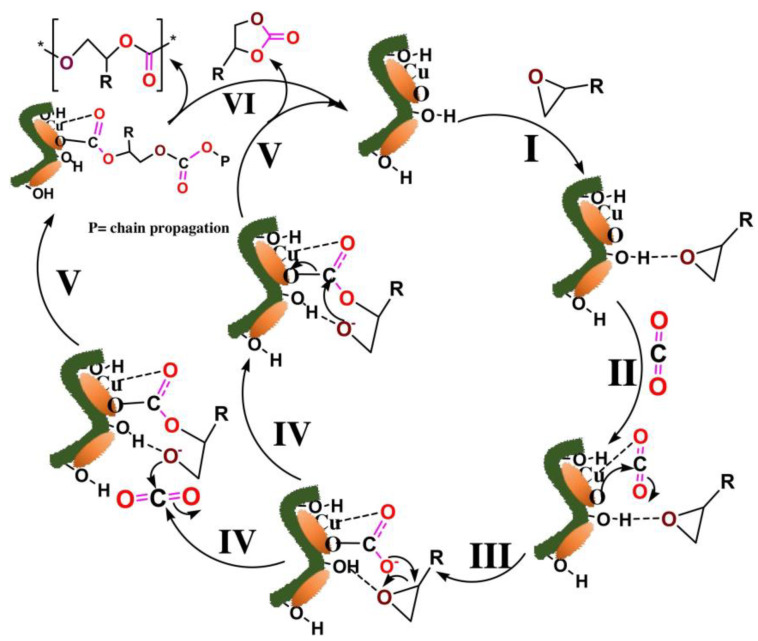
Transformation of epoxides into polycarbonates and cyclic carbonates by use of a carbon dioxide reaction (Adapted with permission from [87]).

As Figure 11 shows, Aggrawal et al. (2021) enhanced producing cyclic carbonates from carbon dioxide and epoxide using this process witha catalyst consisting of a paper matrix of copper oxide nanosheets [87].

The abundant hydroxyl groups present in cellulose make it a versatile material that can serve dual roles in catalysis: as both a co-catalyst and a support for the immobilization of CuO nanoparticles. The hydroxyl groups on the cellulose surface provide reactive sites that can actively participate in catalytic reactions, influencing the catalytic activity and selectivity of the overall system. Under very low carbon dioxide pressure, it was demonstrated that cyclic carbonate could be produced from carbon dioxide and epoxide with an almost 100% yield in under 20 h. Epoxide cycloaddition is the process by which carbon dioxide is activated and adsorbed onto copper oxides base sites. This is because the oxygen in the epoxide attacks the carbon-based carbon in a nucleophilic way, closing the ring and forming thermally stable five-membered cyclic carbonates. In addition, carbonic anhydrase (CA)-loaded calcium cross-linked alginate beads effectively catalyzed the transformation of carbon dioxide into porous calcium carbonate particles by a process induced by carboxymethyl cellulose (CMC) [88]. Protecting this bioactive pigment from heat and light-induced degradation, the porous calcium carbonate particles that were synthesized can be utilized as a carrier for efficient loading of astaxanthin.

### 10.2. Formic Acid and Methanol

Methanol formate, a byproduct of hydrogenation of carbon dioxide, is a typical carbon conversion product. Formaldehyde, dimethyl ether, methyl tertiary butyl ether, acetic acid, and other organic compounds can be made from the converted CO_2_. Additionally, it can be utilized as a fuel for motors and fuel cells. High performance Ru catalysts for the hydrogenation of CO_2_ were prepared by Wang et al. [89] using cellulose and phytate composites (CPCs) as supports. These catalysts were complexed with Ru(III).Hydrotreatment of CO_2_ to formic acid in 1-butyl-3-methylimidazolium acetate was facilitated by the CPC-Ru-complexed catalysts, for example CPC-Ru^−1^, which exhibited enhanced activity.

**Figure 12:**
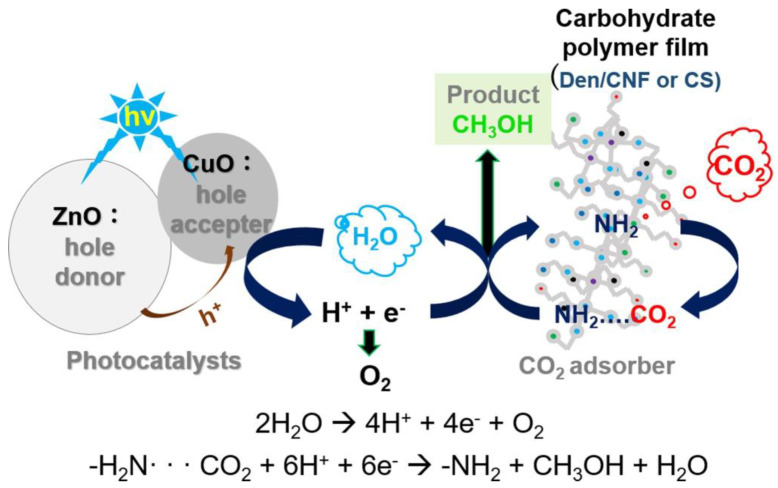
Schematic showing the steps involved in converting CO_2_ into methanol using a CuO/ZnO catalytic membrane (Adapted with permission from [90]).

When it came to the hydrogenation of carbon dioxide to formic acid in 1-butyl-3-methylimidazolium acetate, it demonstrated stronger activity. In 2 h at 120 °C, the initial production rate was 15.21 mol g^−1^ h^−1^, and the selectivity of formic acid was 99%. The conversion was around 1.8 × 10^2^. There is hope for its potential use as a hydrotreatment catalyst for CO_2_ to formic acid thanks to the produced CPC-Ru^−1^ catalyst, which is both inexpensive and highly efficient. CuO/ZnO-Den-TOCNF was made by chemically combining CuO-deposited ZnO with amine-capped poly(amidoamine) dendritic polymers. The amine groups in the dendritic polymers loaded with TOCNF and CS serve as sites for CO_2_adsorption, and the CuO/ZnO acts as a photocatalyst. With a maximum value of 2.16 mmol/g on the CuO/ZnO-Den-TOCNF film, the composite films are able to transform gaseous carbon dioxide into methanol during the photocatalytic conversion process. The oxidation of water to oxygen (O_2_), hydrogen ions (H^+^), and electrons (e^−^) is facilitated by the presence of more copper oxide holes in the CuO/ZnO composite compared to pure zinc oxide, as illustrated in Figure 12 [90].

The composite primarily contains H^+^ and e-ions that come into contact with carbon dioxide and react with dendrimer or chitosan amines to create methanol and a small amount of acetaldehyde. This research presents a carbon conversion method that is both sustainable and safe to the environment.

### 10.3. Carbon Monoxide

**Figure 13:**
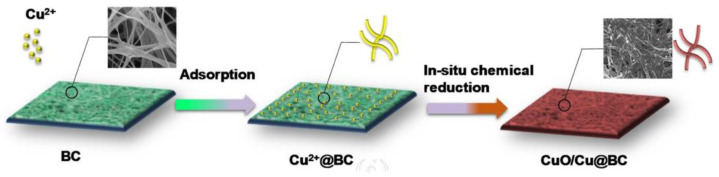
Electrode schematic for CuO/Cu 4:3 @BC (Adapted with permission from [91]).

Apart from methane, formic acid, methanol, inorganic salts, and other products, CO_2_ can also be converted into CO. Zhou et al. (2020) detailed a catalytic electrode made of Bacterial cellulose-supported nanocomposites of Cu and CuO that were scattered throughout [91]. The electrode, called CuO/Cu-4:3@BC, was designed to facilitate the efficient electroreduction of CO_2_ to CO (Figure 13). A CuO/Cu-4:3@BC electrode was produced. Due to the reticulated porous structure of the BC substrate, which allows for faster transport of reactants and products and an increase in the number of electrolyte active sites, the CuO/Cu-4:3@BC electrode exhibited excellent charge transfer performance and a higher current density for CO_2_ reduction. The resultant electrode can create CO with a far-potential efficiency of 53% and maintain activity for 40 h at 490 mV.

**Figure 14:**
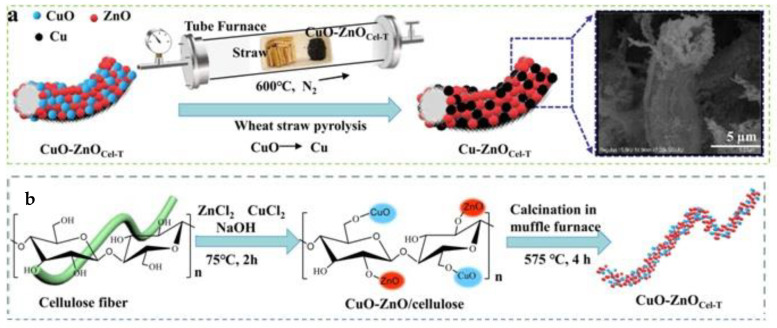
Schematic depicting the steps used to create (**a**) Cu-ZnO_Cel-T_ and (**b**) CuOZnO_Cel-T_ nanocomposites. (Adapted with permission from [92]).

Through the use of cellulose fibers as templates, Shi et al. (2022) created porous ZnO-based catalysts (CuO-ZnO_Cel-T_ and Cu-ZnO_Cel-T_) with varying copper valence states [92]. These catalysts displayed three-dimensional structures and demonstrated outstanding photocatalytic activity as shown in Figure 14a. The CO generation rate of Cu-ZnO_Cel-T_ (30.17 μm mol µg^−1^ h^−1^) is much higher than that of CuO-ZnO_Cel-T_ (8.61 μm mol µg^−1^ h^−1^), even though both catalysts have the same morphology. This suggests that the copper surface plasmon resonance effect promotes the ZnO catalytic activity more than the heterojunction structure of CuO-ZnO_Cel-T_, as Figure 14b shows.

## 11. Challenges and Future Directions

Despite the remarkable strides in developing cellulose-based adsorbents for CO_2_ capture, several technical challenges persist, hindering seamless implementation. One such challenge lies in achieving high adsorption capacities under practical operating conditions. The transition from lab-scale to industrial-scale applications demands a meticulous understanding of the dynamic and varied conditions encountered in real-world scenarios. The fluctuating nature of CO_2_ concentrations in emission streams poses a substantial hurdle. Adsorbents must exhibit robust performance across a spectrum of concentrations and remain effective in the presence of competing gases. Overcoming diffusion limitations and ensuring consistent performance under diverse environmental conditions is a formidable technical challenge that necessitates ongoing research and innovation [93].

Additionally, the issue of adsorbent regeneration efficiency warrants attention. The cyclic stability of cellulose-based materials, particularly in prolonged use, is contingent on their ability to undergo effective regeneration without compromising structural integrity or adsorption performance. The development of efficient regeneration protocols that balance economic viability with minimal environmental impact remains an ongoing technical challenge.

Economic viability is a paramount factor influencing the widespread adoption of cellulose-based adsorbents for CO_2_ capture. While the renewable nature of cellulose offers a sustainable foundation, economic considerations encompass various facets, from raw material costs to production and deployment expenses. The cost-effectiveness of cellulose-based materials is intricately tied to the sourcing of raw materials. The choice of cellulose feedstock, whether sourced from wood, agricultural residues, or other biomass, significantly influences production costs. Furthermore, the development of scalable and sustainable harvesting practices is pivotal to ensuring a consistent supply chain and preventing environmental degradation associated with resource extraction [33,35,42]. The fabrication processes for cellulose-based adsorbents also contribute to economic considerations. The scalability and energy efficiency of manufacturing techniques directly impact production costs. Additionally, the integration of nanotechnological approaches, while enhancing adsorption performance, may introduce complexities in fabrication that need to be carefully balanced against economic feasibility. Another economic consideration involves the lifecycle analysis of cellulose-based adsorbents. Assessing the environmental impact, energy consumption, and end-of-life disposal of these materials is crucial for evaluating their overall sustainability. Balancing economic feasibility with environmental responsibility in a lifecycle context poses a challenge that necessitates a holistic approach to materials development and deployment.

Despite these challenges, the future of cellulose-based materials for CO_2_ capture remains promising. Advancements in nanotechnology, coupled with a deeper understanding of cellulose structure-properties relationships, are expected to address current limitations. Tailoring materials for enhanced selectivity, improved regeneration efficiency, and cost-effective production methods will be pivotal in propelling cellulose-based adsorbents towards widespread adoption. In navigating the challenges and future directions of cellulose-based materials for CO_2_ capture, interdisciplinary collaboration, and sustained research efforts are indispensable. The integration of materials science, nanotechnology, and environmental engineering holds the key to overcoming current limitations and realizing the full potential of cellulose-based adsorbents in mitigating CO_2_ emissions.

## 12. Conclusions

Cellulose stands as a beacon of sustainability in the quest for effective CO_2_ management. Its renewable origin, biodegradability, and inherent versatility offer a unique foundation for the development of advanced materials. The discussion on cellulose-based materials highlighted their capacity to not only capture CO_2_ efficiently but also to serve as a catalyst for the sustainable utilization of captured carbon. The integration of nanotechnological approaches and the development of smart materials showcase the dynamic role cellulose can play in tailoring materials for optimal CO_2_ capture performance. As we transition towards a circular carbon economy, the envisioned breakthroughs in cellulose-based materials hold the potential to revolutionize CO_2_ capture, conversion, and utilization, contributing to a more sustainable and harmonious relationship with our environment. Further research avenues beckon in the exploration of innovative cellulose derivatives, scalable fabrication methods, and the integration of catalytic functionalities. The potential for multifunctional adsorbents capable of capturing and converting CO_2_ into valuable products is an avenue rich with possibilities. The journey towards sustainable CO_2_ management is ongoing, and cellulose-based materials are poised to play a pivotal role. In essence, cellulose, with its intrinsic virtues and transformative potential, emerges as a cornerstone in the relentless pursuit of mitigating CO_2_ emissions and charting a course towards a more sustainable and harmonious coexistence with our planet.

## Data Availability

This is a review article, and no new data were generated or analyzed in this study. All data supporting the findings of this review are available in the cited references.
